# Characterization of Enriched Meat-Based Pâté Manufactured with Oleogels as Fat Substitutes

**DOI:** 10.3390/gels6020017

**Published:** 2020-05-22

**Authors:** Artur J. Martins, José M. Lorenzo, Daniel Franco, Mirian Pateiro, Rubén Domínguez, Paulo E. S. Munekata, Lorenzo M. Pastrana, António A. Vicente, Rosiane L. Cunha, Miguel A. Cerqueira

**Affiliations:** 1 International Iberian Nanotechnology Laboratory, Av. Mestre José Veiga s/n, 4715-330 Braga, Portugal; lorenzo.pastrana@inl.int (L.M.P.); miguel.cerqueira@inl.int (M.A.C.); 2 Centre of Biological Engineering, University of Minho, Campus de Gualtar, 4710-057 Braga, Portugal; avicente@deb.uminho.pt; 3 Centro Tecnológico de la Carne de Galicia, rúa Galicia No. 4, Parque Tecnológico de Galicia, San Cibrao das Viñas, 32900 Ourense, Spain; jmlorenzo@ceteca.net (J.M.L.); danielfranco@ceteca.net (D.F.); mirianpateiro@ceteca.net (M.P.); rubendominguez@ceteca.net (R.D.); pmunekata@gmail.com (P.E.S.M.); 4 Department of Food Engineering, Faculty of Food Engineering, University of Campinas, UNICAMP, CEP, Campinas 13083-862 SP, Brazil; rosiane@unicamp.br

**Keywords:** oleogel, meat product, pâté, functional food, linseed oil, fat replacer, nutrition

## Abstract

Nowadays, one of the strongest factors affecting consumers’ choice at the moment of purchasing food products is their nutritional features. The population is increasingly aware of the diet–health relationship and they are opting for a healthy lifestyle. Concerns with the increasing number of heart-related diseases, which are associated to the consumption of fats, are placing the functional food market in a relevant growth position. Considering that, our goal was to develop, under semi-industrial processing conditions, a healthy meat-based spreadable product (pâté) with reduced fat content through replacement of pork fat by healthier structured oil. Beeswax was used to develop an edible oleogel based on linseed oil with a high content of linolenic acid. A decrease of the hardness and adhesivity was verified for pâtés with oleogel incorporation. Linseed oil inclusion was the main factor leading to an increase of polyunsaturated fatty acids (PUFA) content in pâté samples. A decrease up to 90% in the n-6/n-3 (omega-6/omega-3) ratio can signify a better nutritional value of the obtained pâté samples, which can result in a possible upsurge in omega-3 bioavailability through digestion of these pâtés. This could be an interesting option for the consumption of n-3 polyunsaturated fatty acids, targeting, for example, the reduction of cardiovascular diseases.

## 1. Introduction

Consumers increased their interest into all sorts of diet plans and habits, that go from traditional diets to the unconventional “trendy” ones [1]. Demographics like gender, marital status, education level and location are amongst some of the main characteristics of the focus groups for the development of a new product [2]. From the industry standpoint, product offering is based on different aspects, being some of the drivers of this process the increased consumer awareness on the damaging effects of unbalanced food consumption and their desire to have a healthier diet and lifestyle [3,4,5]. However, for the industry, new healthier products should not induce considerable modifications in the manufacturing process and the increase of production costs, and therefore not influence the availability of such new products to the consumers.

Spreadable liver pâtés are very popular food products [6,7]. These are pastes normally produced with pork liver, precooked pork backfat, water, sodium caseinate and small quantities of other additives [8], in which animal sources may vary depending on the country of production. These products are consumed all over the world and are usually valued by consumers due to their increased sensory qualities, such as intense flavour and appellative spreadable texture [9]. Pâtés are regarded as food products with high animal fat content, ranging from 35% to 50% [10]. Because of that, and since the consumers’ demands for healthier foods are intensifying, some alternatives have been proposed to make these products healthier, particularly those aiming at suppressing hard fat amounts [3,6,11,12,13,14]. These include the hydrogenation of fatty acids (hydrogenated oils or natural oils with high content in saturated fats to be used as shortenings), interesterification (blends of oils with a high saturated fatty acids content or mixtures of hydrogenated solid fats with liquid unsaturated edible oils) and blending of fats (high melting triglycerides used for fractionation and fat blending) [15]. However, as a result of the guidelines from the Food and Drug Administration (FDA), partially hydrogenated oils are no longer recognized as GRAS (Generally Recognized as Safe) since July of 2018. Western countries’ diet is still poor on n-3 polyunsaturated fatty acids (PUFA) and recent data demonstrated that the n-3:n-6 PUFAs ratio is of 1:15-20. These values are very lopsided if we compare them to the diet of our ancestors, which was estimated to have a ratio of 1:1 [16,17]. According to experts on the subject, the recommended ratio is around 2:1 [18,19]. Recently, nutritional directives encourage the consumption of increased dosages of n-3 PUFA, in order to reduce the risk of cardiovascular diseases, stroke, heart failure, and atrial fibrillation in a significant manner [16]. Works focused on meat-based pâtés reformulation with fat consumption in mind and show a different number of approaches. Lorenzo et al. [20] studied the physic and chemical profile of pâtés, based on the nature and fat content of the meat used in their formulation, concluding that distinct sources of fat influence the final properties of pâtés, namely texture and n-6/n-3 ratio. Some studies report fat substitution in meat products, however regarding pâté and the use of healthy structured oils, studies are scarce. Delgado-Pando et al. studied the application of different oil combinations (vegetable and fish oils) with konjac hydrogels to produce low fat liver pâtés with fat and saturated fatty acids contents of 50% and 33 %, respectively [21]. The direct replacement of animal fat by oil emulsion [6,7,22] or encapsulated oils [5] in pâté reformulation strategy were also reported by multiple researchers. As a general conclusion, the nutritional characteristics of reformulated pâtés improved as the animal fat was replaced by vegetable/marine oils. Oleogels are composed by a structured oil phase, which makes them suitable to be easily incorporated as fat replacers during the pâté processing stage. Barbut et al. also demonstrated the feasibility of incorporating ethylcellulose-based oleogels with specific characteristics in pâté, achieving promising end results in terms of comparable textural and sensorial properties with the control samples [23]. Gomes-Estaca et al. recently reported on the incorporation of ethylcellulose and beeswax oleogels using oil blends in pâtés, showing promising physicochemical and sensorial properties [24]. The main challenge resides in not compromising some main characteristics, namely water, fat binding and consequently pâté cohesiveness, which will influence sensorial features of this particular food product. Regularly used fats serve as important sources of structure and taste for meat-based products, since assembled networks of crystalline triacylglycerols are responsible for mouth-feel and textural experiences.

Depending on the gelator used to structure the oil, the morphological characteristics of the gels can be altered (e.g., melting point, hardness, colour, stickiness). It is also possible to promote changes at the molecular level, by modifying the fatty acid profiles, which can be achieved using specific oil phases in oleogel development [25].

With the aforementioned in mind, the main objective of this work was to study the effect of pork back fat replacement on the physicochemical and nutritional properties of pâté, using linseed beeswax-based oleogel as a suitable alternative for saturated fat reduction on pâté formulation. Beeswax, like other waxes, is a versatile gelator, capable of enduring gelation using low concentrations in very different types of oils [26,27,28]. Linseed oil was selected because of its rich composition n-3 alpha-linolenic fatty acid, that has been identified as an important cardioprotective agent [29]. The incorporation of linseed oil in the final spreadable product will be responsible for an imbalance of the n-6/n-3 ratio, favouring an increase in omega-3 fraction. Selected fractions (30% and 60%) of fat were replaced by linseed beeswax-based oleogels and textural and physicochemical properties were evaluated in order to understand the impact of the oleogel on the pâté. Wide-ranging consumer and acceptance sensorial tests were also performed.

## 2. Results and Discussion

### 2.1. Physicochemical Composition of Elaborated Pâtés

The incorporation of the beeswax oleogel with linseed oil, as the lipid phase affected the physicochemical properties (Table 1) and fatty acid profile of pâté (Table 2).

The pH values reported in the present research agree with those reported in previous studies [6,7]. The P-60 samples showed the lowest values and a progressive and continuous decreased of pH values were found as the animal fat was replaced by oleogel. This fact could be because the oleogel was more acid than animal fat, and then a significant reduction of pH values was observed in samples with 60% replacement. This fact was also reported by other authors that use oleogel with beeswax-linseed oil in dry-fermented sausages [30]. 

Proximate compositions of pâté samples were significantly influenced by oleogel incorporation. The replacement of animal fat by oleogel resulted in a progressive increase in moisture content and a significant decrease in protein and ash content. In contrast, a previous study did not find differences in the proximate composition of pork pâté reformulated with partial and total fat replacement by olive oil [6]. In the present study, the moisture contents (50–52%) agree with the results reported by other authors in pâté, who reported values of 51–52% in pâté reformulated with olive oil [6], 50–53% in pâté reformulated with fish oil [7,22] and 52–54% in deer pâté with replacement of animal fat by encapsulated vegetable oils [5]. Similarly, the content of ash also agree with those reported in previous studies in which ash content of control and reformulated pâtés ranged between 3% and 4% [5,22]. 

On the other hand, the higher protein content found in control samples than in the P-30 and P-60 could be related with the fact that pork backfat contains about 9% of protein [5]. Thus, the partial replacement of backfat by oleogel (without protein) resulted in a significant reduction in protein content. The values reported in other studies made with pâté [22] and frankfurter type sausages [13] agree with our data. In contrast, other authors reported higher protein values (18–24%) in reformulated pâtés [5,6]. These differences are related with the fat/oil content used in the pâté formulation.

Regarding fat content, the replacement of 30% of animal fat resulted in a slight fat reduction (*p* > 0.05), while the samples of P-60 showed a significant reduction (20.55% vs. 27.16%). From nutritional point of view, this reduction is a positive goal. This fact could be related with the different lipid content between pork backfat and oleogel made with a mixture of beeswax and linseed. In the literature there is controversy in the results. The strategy used for fat substitution has a great influence on the result. Thus, the works that use the encapsulation of oils observe a significant reduction in the fat content of frankfurter type sausages [13] and pâté [5]. However, the application of oil directly to the meat mass of the pâté results in a significant increase in fat [6,22]. In previous studies made with oleogels, the use of oleogel with beeswax-linseed oil in frankfurter sausages [31] and dry-fermented sausages [30] did not modify fat content.

In the P-CO samples, the fatty acid profile was the same as those reported in pork backfat [5]. The major fatty acid was C18:1n-9 followed by C16:0, C18:2n-6 and C18:0. In contrast, the partial replacement of pork backfat by oleogel resulted in a progressive and significant increase in fatty acids from linseed (mainly C18:3n-3) and a proportional reduction in the other fatty acids. In this regard, fatty acid profile in P-30 samples was C18:1n-9>C18:2n-6=C16:0>C18:3n-3, while in the P-60 samples the major fatty acid was C18:3n-3, followed by C18:1n-9, C18:2n-6 and C16:0. 

It is well known that linseed oils have high amounts of C18:3n-3 (about 55% of total fatty acids). Thus, the greatest changes observed in this fatty acid among the samples reflected the fatty acid composition of the oil used in the present study. Similar results were reported in a recent work, in which authors replaced pork backfat by encapsulated linseed oil in deer pâté [5]. These authors, as occurs in the present research, found that the content of C18:3n-3 suffered a dramatic increase when linseed oil was included in the formulation.

**Figure 1:**
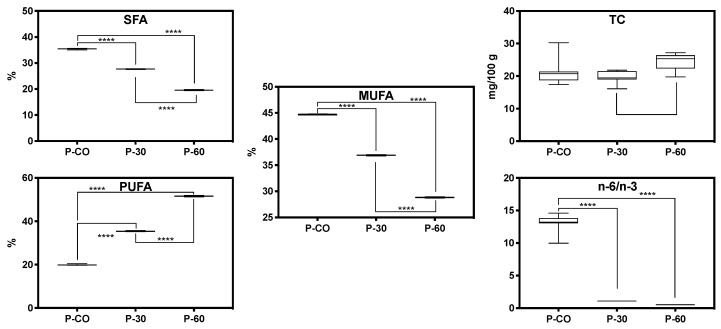
SFA—Saturated fatty acids; MUFA—Monounsaturated fatty acids; PUFA—Polyunsaturated fatty acids; TC—Total cholesterol; n-6/n-3—ratio of omega-6/omega-3.

Moreover, from a nutritional point of view, the inclusion of linseed oleogel had an important influence in the fatty acids profile. The content of saturated fatty acids (SFA) decreased as the pork backfat was replaced (from 35.4% in control samples to 27.7% and 19.6% in P-30 and P-60, respectively) (Figure 1). This reduction is related with the significant decrease in the content of the most important saturated fatty acids: C16:0, C18:0 and C14:0. Additionally, except for the amounts of C20:0, C22:0 and C24:0 that slightly increased with the oleogel inclusion, the other individual SFA decreased as the pork backfat was replaced by oleogel. Similarly, the content of monounsaturated fatty acids (MUFA) also suffered a significant decrease with the inclusion of oleogel. The MUFA content of P-CO (44.7%) diminished to 36.9% in P-30 and 28.8% in P-60. In this case, all individual MUFA showed a progressive and significant reduction in their amounts with the partial replacement of pork backfat. However, the major effect was visible in the content of C18:1n-9, C18:1n-7 and C16:1n-7. In previous researches, the replacement of animal fat by oils with high content of polyunsaturated fatty acids (PUFA) also resulted in a significant reduction in total and individual SFA and MUFA [5,22].

Finally, as expected the amount of PUFA increased dramatically with the addition of oleogel (19.9% vs. 35.4% and 54.6% in P-CO, P-30 and P-60, respectively). Regarding PUFA, individual n-6 PUFA, such as C18:2n-6, C20:4n-6, C20:2n-6 or C20:3n-6, showed similar values or suffered a slight decrease with the animal fat replacement. In fact, the total n-6 content was very similar among batches (18.8% in P-CO and P-30 and 17.6% in P-60). In contrast, the amount of n-3 PUFA increased from 1.43% in P-CO to 16.8% in P-30 and 33.8% in P-60. This increase is directly related with the higher amounts of C18:3n-3 in the samples reformulated with oleogel. Finally, due to these results, the n-6/n-3 ratio decreased from 13 in P-CO to 1.09 in P-30 and 0.51 in P-60. Our findings agree with those reported by other authors. In this sense, the use of oleogel in dry-fermented sausages showed no significant differences in the n-6 content, while the amount of n-3 PUFA (and C18:3n-3) increased significantly and the n-6/n-3 ratio decreased [30]. The same results were reported in burgers [32,33] and pâté [5] reformulated with linseed oil. Thus, taking into account the results obtained in the P-30 and P-60 pâtés, they can be claimed as “reduced saturated fat” according to the European Regulation [34]. Additionally, the reformulated pâté from these batches can be also claimed as “high content of omega-3” because both presented higher amounts than the minimum value (0.6 g C18:3n-3/100 g of product) reported in the Regulation [34]. 

Finally, the results revealed that the pork fat replacement by linseed-oleogel resulted in a decrease of 21% and 55% of SFA in P-30 and P-60 samples, respectively. According to the World Health Organization, it is known that the diets rich in fat (particularly saturated fat) can increase the risk related to developing coronary heart disease [35]. Additionally, an adequate and balanced PUFA intake must be consumed to prevent multiple diseases. The European Food Safety Authority—EFSA [36] reported that there are not sufficient data to define a precise fat intake to SFA, MUFA, PUFA or n-6/n-3 ratio. However, several international authorities such as EFSA, FAO and USDA recommended that SFAs intake should be as low as possible [36] or less than 10% of calories (2000 or 2500 calorie diet) by replacing them with MUFAs and PUFAs [37,38]. On the other hand, according to FAO nutritional recommendations [38], the n-6/n-3 ratio should be less than 4.0. Nutritional values obtained in reformulated pâtés with linseed-oleogel satisfied advice proposed by the international authorities (EFSA, FAO and USDA) due to significant values (*p* < 0.001) of SFA substitution by PUFA and lower levels than those ratios recommended (<4.0) were achieved.

On the other hand, the cholesterol content varied between 19.7 mg/100 g (P-30) and 24.4 mg/100 g (P-60). The control samples showed intermediate values (21.4 mg/100 g). Our values are lower than those reported by other authors in deer pâté reformulated with different encapsulated vegetable oils (between 27.8 and 39.2 mg/100 g) [5]. In other study, in which pork fat was partially replaced by olive oil also reported higher cholesterol values (26.5-35.2 mg/100 g) than those found in the present research [6]. 

### 2.2. Textural and Colour Measurements

**Figure 2:**
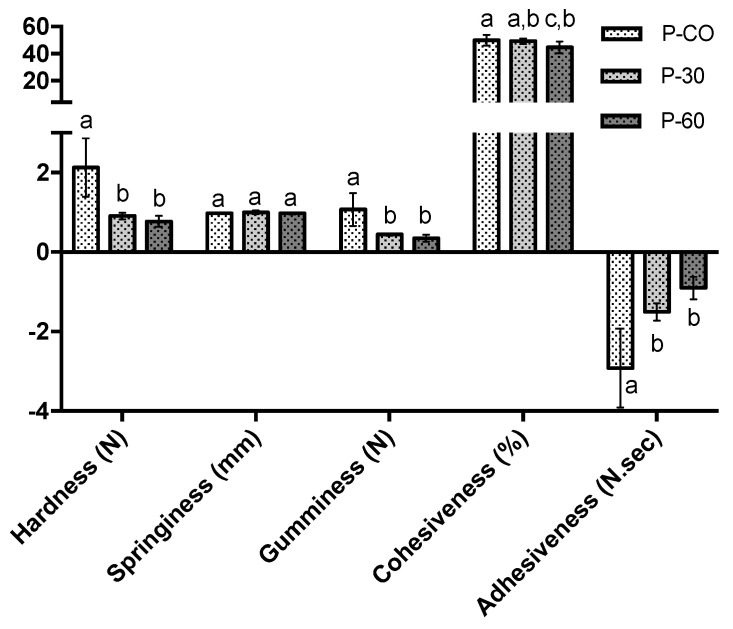
Texture (hardness; springiness; cohesiveness; gumminess and adhesiveness) for P-CO, P-30 and P-60 pâté samples, as a result of texture profile analysis (TPA) analysis. ^a–c^ Different letters (in the same parameter) mean that samples are statistically different (α = 0.05).

Figure 2 shows the influence of oleogel incorporation in pâté textural properties. Oleogel incorporation in pâté composition produced a significant decrease in the hardness values of pâté samples. These results were expected because beeswax−based oleogels are among the less shear resistant oleogel structures, as verified in previous works [27,28]. The softer consistency, demonstrated by P-30 and P-60, is a result of the decrease in saturated fats and the increase in polyunsaturated fats when pork back fat was replaced by oleogels. This behaviour is in accordance to the ones reported in the literature when sources of saturated fat were replaced by unsaturated ones in pâté formulations; it was also noted that the adipocyte structure of back fat tissue remains intact after the manufacture contributing to the higher consistency of the control batches [6,39]. The adhesiveness was also influenced by the oleogel incorporation as a consequence of a more disintegrated macrostructural arrangement of the final pâté samples. This oleogel formulation, with 8% (*w*/*w*) of gelator, was not able to convey the same meat-binding properties as the pork backfat present in control samples, following the mechanical movements that were applied during pâté processing (grinding). The reduced adhesiveness obtained for P−30 and P−60 samples is a consequence of less binding activity provided by the oleogel. In addition, when comparing with the control sample, P-30 and P-60 revealed less gumminess after the shearing process resulting in a decrease of the sticky response from the samples. There were no differences for the springiness parameter in all samples. Differences between the P-CO and P-60 were the ones observed for the cohesiveness response, which measures how a product withstands a second deformation relative to the deformation observed in the first one. Texture values obtained for P−30 and P−60 (for all parameters) were similar, even when using the double of the oleogel mass. 

One of the alternatives to reach the values of hardness and adhesiveness of the control pâté samples, could be the use of a beeswax-based oleogel with increasing gelator concentration, hence increasing textural parameters [40]. This could increase the overall hardness properties of pâté samples but only at relatively higher concentrations in order to withstand shearing effects during processing. However, this is a formulation that could alter the consumer acceptance and preference for the product, therefore this particular aspect needs further work.

The addition of the oleogel in pâtés formulation was responsible for significant changes in the colour parameters of P-30 and P-60 samples. The colour values found by us were similar to those reported in previous studies made in pâtés [5,6]. Table 3 shows the CIE L*a*b* colourimetric coordinates, as well as the RGB conversion (performed using Matlab software) and the observed colour for pasteurized pâté samples. As the amount of added oleogel increases, the lightness values decrease significantly and the b* coordinate shows increased values; as a consequence of that, pâté colour changes towards a richer yellowish tone. This yellow tonality is a contribution from the oil phase and also from the presence of (opaque characteristic) beeswax.

### 2.3. Sensorial Tests

#### 2.3.1. Acceptance Results

**Figure 3:**
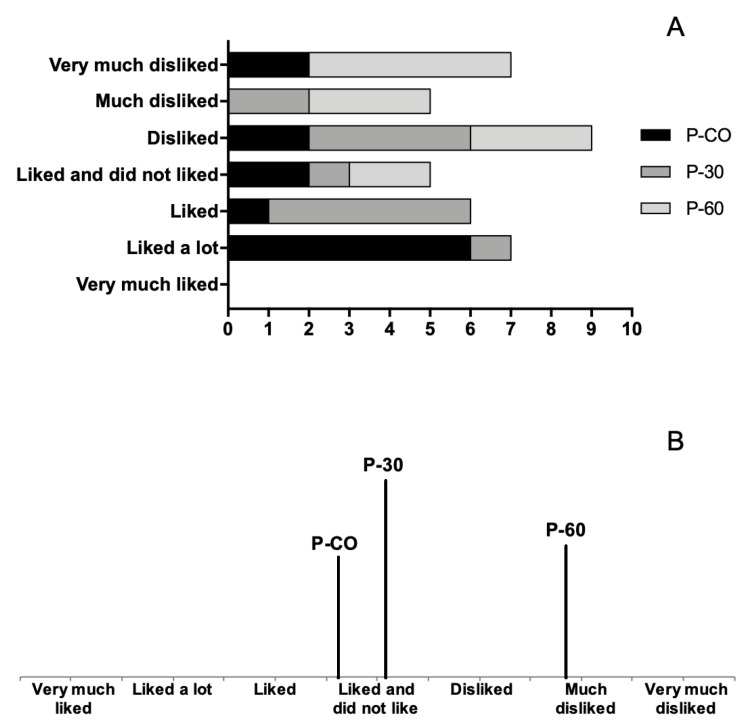
(**A**) Number of preference selections made for every pâté sample; (**B**) global acceptance overall results; (P-CO: control sample; P-30 and P-60: samples with 30% and 60% of pork fat replacement, respectively).

The results of the sensorial tests were conclusive in terms of the preference and acceptance of the panellists concerning the tested pâté samples. After the tastings, collected data analysis indicated that the control sample (P-CO) and P-30 were the most valued samples, with a combined score for “liked” and “liked a lot” of 7 and 6, respectively. The overall score for the P-30 sample remains positively interesting if we consider that the results are not far from P-CO. In contrast, the global acceptance score for P-60 is distant from the other two samples and was classified as “much disliked” and “very much disliked” by 8 out of the 13 panellists (see Figure 3A), which revealed the displeasing feedback gathered. The global acceptance score results from the grades given by the panellists in their evaluation are shown in Figure 3B. The score for P-CO, P-30 and P-60 was of 3.62, 4.08 and 5.85, respectively. It is safe to say that even the control sample did not please some panellists as is visible in the overall score displayed by in Figure 3B.

#### 2.3.2. Preference Results

**Figure 4:**
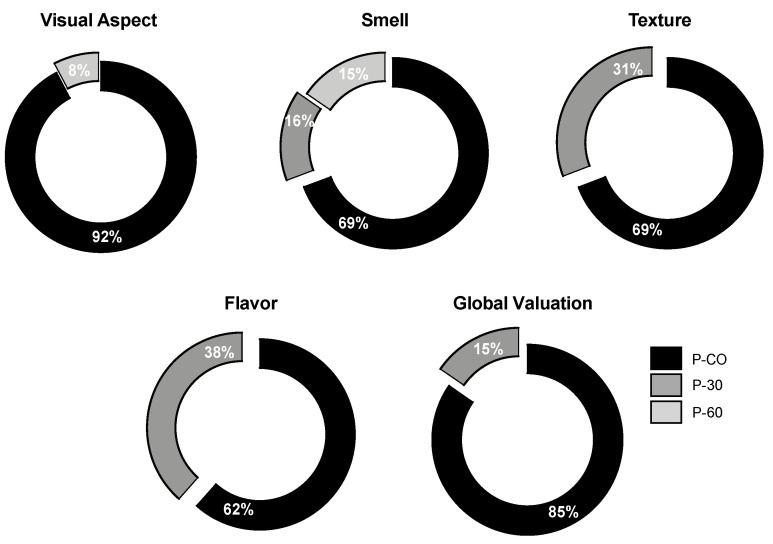
Preference score for each of the parameters showed in percentage; (P-CO: control sample; P-30 and P-60: samples with 30% and 60% of pork fat replacement, respectively).

The evaluation of the pâtés was also performed considering the following properties: the visual aspect of pâté, smell, tenderness, juiciness, taste and global valuation. The preferred pâté sample in each of the tested parameters is shown in Figure 4. P-CO sample was selected as the one with the best visual aspect. A total of 12 (92%) participants selected P-CO samples. The best global valuation was obtained for the P-CO sample, as classified by 11 (85%) participants, followed by 2 (15%) that selected the P-30 and no one in the panel selected the P-60. This means that the control sample (P-CO) is the number one preferred sample in all the parameters. For three of the five surveyed parameters, P-60 was not even considered by the members of the panel.

The most unbalanced results were the visual aspect of the pâté and their global valuation from the panellist group with 92% and 62% selection value, respectively. A relation can be established between the amount of linseed oil, with its strong aroma and the flavour of the pâté. It is important to mention that the P-60 sample was not even considered by any of the panellists. The same happened for the texture classification, which can be related to the lesser adhesiveness recorded for P-60 in the texture measurements (as presented in Section 2.2). The textural parameters are extremely important for the visual aspect classification, where only 8 % of the evaluators selected the P-30 as the best. This is a result of the less hardness and gumminess exhibited (P-60 was not even selected for this category).

The Friedman Test was useful to understand the disparities in terms of the panellists’ sensitivity regarding the three sets of pâté samples. This analysis registered the statistical differences between the control sample and the set of samples with the highest level of fat substitution (P-60), for all the parameters. Values obtained with the Friedman rank sum test are presented in Table 4. This is an indication that parameter valuation exhibited non-significant differences between P-CO and the P-30 set of samples. However, some improvements can be done, such as an approximation of the P-60 composition to the control so that satisfactory results can be achieved.

## 3. Conclusions

The introduction of a healthier solution, capable of replacing saturated fat, in pâté formulation, was demonstrated in this work. The concentration of beeswax used was able to successfully induce the gelation of linseed oil, and the resultant oleogel was introduced in the pâté elaboration process, without additional unitary stages that could eventually compromise an already well-established process. The linseed oil, added in the form of an oleogel, was responsible for the increase in polyunsaturated fatty acids (PUFAs). As a result, the omega-3 intake potential of the pâtés with oleogel incorporated was increased, therefore adding value to a product that is not normally seen as a source of health benefits. In fact, the n-6/n-3 ratio demonstrated that the pork backfat replacement by oleogel resulted in healthier pâté in comparison with control. Pâté mechanical characteristics were accessed and, despite differences were found in some of the parameters (e.g., hardness and cohesiveness), these are not enough to limit the utilization of such technology in this type of application. Nevertheless, tailoring the properties that are associated with oleogels must be further explored in order to close the observed gap, in terms of consumer preference, between the control and the oleogel-based pâté samples.

This work opens the possibility of tailoring the fatty acid profile of meat-based products (pâté in this case), configuring very promising outcomes for a tailor-made product in the near future. This shows the potentialities of the replacement of fats in food composition by oleogels in order to tailor nutrition in meat-based food products. Future research should comprise digestive studies of these pâtés in order to further understand the bioaccessibility and bioavailability of omega-3. The capability of enlarging the portfolio of meat industries, with a versatile approach towards functionality in common “off the shelf” food products, makes this approach very interesting to respond to the most recent consumer habits towards a healthier diet.

## 4. Materials and Methods

### 4.1. Raw Materials and Oleogels Production

Pâté formulation comprised the formation of an emulsion constituted by pork subcutaneous fat, sodium caseinate, cold water, portions of lean pork meat, chopped liver, salt and water. For the production of beeswax-based oleogel a commercial linseed oil (Vitaquell^®^, Hamburg, Germany) with 72% polyunsaturated (approx. 55% of α-linoleic), 19% monounsaturated and 9% saturated fatty acids was used as the oil phase. Oleogels with 8% (*w*/*w*) of gelator were produced for all the fat replacement experiments. Beeswax was dispersed in linseed oil under stirring at 80 °C (above wax melting point) for at least 30 min. After that period of time, the gels were left cooling at room temperature until full gel formation, for at least 48 h.

### 4.2. Pâté Elaboration

The ingredients used for pâté preparation are described in Table 5. 

**Figure 5:**
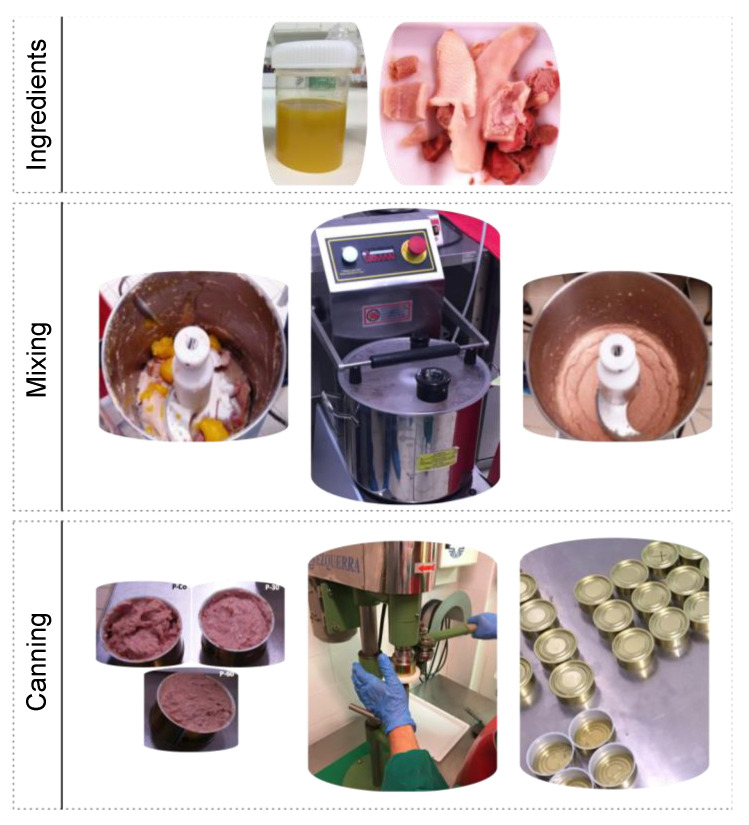
Main stages of pâté production.

Pâté elaboration consisted in mixing all the ingredients (Table 5) using an automatized mincer (C15 cutter from Sirman, Italy) for 10 min in order to obtain a homogeneous fine paste (Figure 5). For samples P-30 and P-60 the necessary quantity of beeswax-based oleogel was added in the process as a substitute for pork subcutaneous fat. All samples were canned and then subjected to pasteurization and cooling for proper preserving conditions before all subsequent tests were performed.

### 4.3. Physicochemical Composition of Elaborated Pâtés

Moisture [41], protein [42] and ash [43] were quantified in accordance to the ISO recommended standards. Total fat was extracted according to the AOCS Official Procedure Am 5−04 [44]. The pH of the pâté samples was obtained using a digital portable pH−meter (HI 99163, Hanna Instruments, Eibar, Spain) that was equipped with a glass penetration probe. For the quantification of total cholesterol (TC), saponification, extraction, and the identification was performed using a high performance liquid chromatography (HPLC) in accordance with Domínguez et al. [45].

#### 4.3.1. Fatty Acid Composition

Chloroform/methanol mixture was used to extract the fat from 10 g according to Barros et al. [14]. Lipids were evaporated under nitrogen in a water batch (50 °C) and stored at −80 °C until analysis by preparation of fatty acid methyl esters (FAMEs). Lipids’ transesterification was performed using sodium methoxide (0.5 N) and H_2_SO_4_-Methanol solution in 50 mg of the extracted lipids, forming FAMEs. FAMEs separation and quantification was carried out using a GC−Agilent 7890 gas chromatograph (Agilent Technologies Spain, S.L., Madrid, Spain) following the chromatographic conditions described by Barros et al. [14].

#### 4.3.2. Textural and Colour Analysis

A TA-XT.plus texture Analyzer (Stable Micro Systems, Vienna Court, UK) was used according to Bourne et al. [46]. The penetration tests were carried out at room temperature (20 ± 2 °C) and performed with a 6 mm diameter penetration probe (with 5 kg charge cell) at a velocity of 0.8 mm·s^−1^ with a travelling distance of 8 mm. Hardness (N), springiness (mm), gumminess (N), and adhesiveness (N·s) values were obtained using the available computer software (TEE32 Exponent 4.0.12. Stable Micro Systems, Vienna Court, UK). Colour was measured, after the canning procedure, using a portable colorimeter (Konica Minolta CM−600d, Osaka, Japan) equipped with pulsed xenon arc lamp, 0 degrees viewing angle geometry and 8 mm aperture size, to estimate pâté colour in the CIELAB space: lightness, (L*); redness, (a*); yellowness, (b*) that were afterwards converted to RGB coordinates. Three different points of each sample in homogeneous and representative areas.

### 4.4. Consumer Sensorial Evaluation

All the consumer sensorial evaluation tests were performed under the UNE-EN ISO 8589:2010 normative, in a room equipped with single tasting booths under white light. These studies were carried out with the main objective of evaluating consumers’ acceptance and preference regarding the different pâté samples. The sensorial evaluation panel was composed by 13 tasters recruited among the Meat Technology Centre. The acceptance test consisted of a sensorial evaluation of a number of attributes right after tasting. A 7-point scale (very much liked; liked a lot; liked; liked and did not liked; disliked; much disliked; very much disliked) was used by each member of the panel to measure the mentioned parameters. Differently, the preference test was based on a single classification, for each pâté sample, in line with the following sensorial attributes: visual aspect; smell; texture; flavour; global evaluation. In order to prevent the impact of the so-called “carry over effect”, sample evaluations were conducted using different sample distribution for each member of the panel. All sensorial tests results were statistically treated by means of the non-parametric ranked sum Friedman test, commonly used to attest differences between a number of related samples, when the same parameter has been measured under different conditions on the same subjects. The grade of 1 was given to the preferred sample, grades 2 and 3 were given, respectively, for the second and the third most preferred samples.

### 4.5. Statistical Analysis

A total of 24 samples (3 pâté formulations X 8 replicates of each formulation) were analysed in the present research. All the statistical analyses were performed using Analysis of Variance, Tukey’s mean comparison test (* for *p* < 0.05; ** for *p* < 0.01; *** for *p* < 0.001; **** for *p* < 0.0001) from results conveyed as an average and standard deviation (GraphPad Software, Inc. USA). For the sensorial results, the Friedman statistical test was used in order to understand if there were statistically significant differences among the classifications given by the panel (α = 0.05).

## Figures and Tables

**Table 1:** pH and chemical composition of all sets of pasteurized pâté samples; (P-CO: control sample; P-30 and P-60: samples with 30% and 60% of pork fat replacement, respectively).

Parameter	P-CO	P-30	P-60
**pH**	6.45 ± 0.03 ^a^	6.38 ± 0.2 ^a,b^	6.29 ± 0.01 ^b^
**Protein (%)**	13.12 ± 0.72 ^a^	10.75 ± 0.36 ^b^	11.12 ± 0.71 ^b^
**Moisture (%)**	50.79 ± 1.53 ^a^	51.39 ± 1.06 ^a,b^	52.83 ± 1.53 ^b^
**Fat (%)**	27.16 ± 4.41 ^a^	26.34 ± 2.43 ^a^	20.55 ± 1.86 ^b^
**Ash (%)**	3.00 ± 0.10 ^a^	2.70 ± 0.12 ^b^	2.77 ± 0.05 ^b^

^a,b^ Different letters (in the same line) mean that samples are statistically different (α = 0.05).

**Table 2:** Fatty acid composition of pâté samples (P-CO: control sample; P-30 and P-60: samples with 30% and 60% of pork fat replacement, respectively).

Fatty Acids	P-CO	P-30	P-60
**C10:0**	0.058 ± 0.004 ^c^	0.040 ± 0.002 ^b^	0.021 ± 0.001 ^a^
**C12:0**	0.067 ± 0.001 ^c^	0.049 ± 0.002 ^b^	0.025 ± 0.000 ^a^
**C14:0**	1.19 ± 0.002 ^c^	0.878 ± 0.002 ^b^	0.428 ± 0.001 ^a^
**C15:0**	0.085 ± 0.001 ^c^	0.055 ± 0.000 ^b^	0.044 ± 0.000 ^a^
**C16:0**	21.97 ± 0.079 ^c^	17.14 ± 0.059 ^b^	11.15 ± 0.078 ^a^
**C17:0**	0.472 ± 0.002 ^c^	0.312 ± 0.002 ^b^	0.286 ± 0.004 ^a^
**C18:0**	11.34 ± 0.112 ^c^	8.79 ± 0.047 ^b^	7.04 ± 0.074 ^a^
**C20:0**	0.193 ± 0.003 ^a^	0.215 ± 0.001 ^b^	0.220 ± 0.003 ^c^
**C22:0**	n.d. ^a^	0.063 ± 0.001 ^b^	0.116 ± 0.009 ^c^
**C24:0**	n.d. ^a^	0.127 ± 0.004 ^b^	0.217 ± 0.015 ^c^
**C16:1n-7**	2.12 ± 0.009 ^c^	1.61 ± 0.018 ^b^	0.688 ± 0.005 ^a^
**C17:1n-7**	0.347 ± 0.002 ^c^	0.205 ± 0.002 ^b^	0.148 ± 0.003 ^a^
**11t-C18:1**	0.268 ± 0.001 ^c^	0.203 ± 0.002 ^b^	0.116 ± 0.002 ^a^
**C18:1n-7**	2.98 ± 0.009 ^c^	2.32 ± 0.011 ^b^	1.59 ± 0.009 ^a^
**C18:1n-9**	38.04 ± 0.097 ^c^	31.92 ± 0.083 ^b^	26.19 ± 0.094 ^a^
**C20:1n-9**	0.947 ± 0.004 ^c^	0.657 ± 0.003 ^b^	0.039 ± 0.016 ^a^
**9t,11t-C18:2**	0.024 ± 0.001 ^c^	0.022 ± 0.002 ^b^	0.014 ± 0.000 ^a^
**C18:2n-6**	16.77 ± 0.048 ^b^	17.16 ± 0.022 ^c^	16.58 ± 0.007 ^a^
**C20:2n-6**	0.693 ± 0.002 ^c^	0.499 ± 0.002 ^b^	0.265 ± 0.002 ^a^
**C18:3n-3**	1.15 ± 0.193 ^a^	16.63 ± 0.181 ^b^	33.67 ± 0.269 ^c^
**C18:3n-6**	0.056 ± 0.001 ^a^	0.106 ± 0.001 ^b^	0.162 ± 0.002 ^c^
**9c,11t-C18:2 (CLA)**	0.107 ± 0.001 ^a^	0.147 ± 0.001 ^b^	0.210 ± 0.003 ^c^
**C20:3n-6**	0.155 ± 0.002 ^c^	0.129 ± 0.003 ^b^	0.107 ± 0.005 ^a^
**C20:4n-6**	0.130 ± 0.013 ^c^	0.096 ± 0.009 ^b^	0.069 ± 0.016 ^a^
**C22:5n-3**	0.728 ± 0.001 ^c^	0.536 ± 0.002 ^b^	0.492 ± 0.002 ^a^

CLA meaning Conjugated Linoleic Acid; ^a–c^ Different letters (in the same line) mean that samples are statistically different (α = 0.05).

**Table 3:** Colour parameters (L*, a*, b*) with RGB conversion of pâté samples (P-CO: control sample; P-30 and P-60: samples with 30% and 60% of pork fat replacement, respectively).

Sample	L*	a*	b*	R	G	B	Actual Colour
**P-CO**	63.81 ± 4.04 ^a^	5.23 ± 0.55 ^a^	20.88 ± 2.06 ^a^	174	151	118	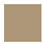
**P-30**	63.33 ± 5.25 ^a^	4.48 ± 0.54 ^b^	23.82 ± 1.09 ^b^	172	150	111	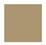
**P-60**	59.77 ± 2.75 ^a^	5.66 ± 0.39 ^a^	28.29 ± 2.79 ^c^	166	140	94	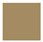

^a–c^ Different letters (in the same line) mean that samples are statistically different (α = 0.05).

**Table 4:** Friedman test results using a valuation of 1, 2 or 3 for the preferred sample, second preferred sample and the third preferred sample, respectively. F(test) > F(*α* = 0.05).

Parameters	P-CO	P-30	P-60
**Visual Aspect**	**15 ^a^**	**27 ^a,b^**	**36 ^b^**
**Smell**	**19 ^a^**	**26 ^a,b^**	**33 ^b^**
**Texture**	**18 ^a^**	**23 ^a,b^**	**37 ^b^**
**Flavour**	**19 ^a^**	**22 ^a^**	**37 ^b^**
**Global valuation**	**15 ^a^**	**25 ^a,b^**	**38 ^b^**

^a,b^ Different letters (in the same line) mean that samples are statistically different (α = 0.05).

**Table 5:** pH and chemical composition of all sets of pasteurized pâté samples; (P-CO: control sample; P-30 and P-60: samples with 30% and 60% of pork fat replacement, respectively).

Raw Materials (Wt%)	P−Co	P−30	P−60
Pork subcutaneous fat	40	28	16
Linseed oleogel	/	12	24
Sodium caseinate	2	2	2
Cold Water	23	23	23
Lean meat	15	15	15
Liver	18	18	18
Sodium chloride	2	2	2
